# Diet Quality and Cognitive Performance in Australian Adults Aged 55–85 Years: A Cross-Sectional Analysis of the Hunter Community Study Cohort

**DOI:** 10.3390/nu13030909

**Published:** 2021-03-11

**Authors:** Pui Fung Li, Mark A. McEvoy, Sharmaine McKiernan, Peter W. Schofield, Lesley K. MacDonald-Wicks, Amanda J. Patterson

**Affiliations:** 1 Department of Paediatrics, Faculty of Medicine, The Chinese University of Hong Kong, Hong Kong, China; puifungli@cuhk.edu.hk; 2 School of Health Sciences, College of Health, Medicine and Wellbeing, University of Newcastle, University Drive, Callaghan, NSW 2308, Australia; sharmaine.mckiernan@newcastle.edu.au (S.M.); lesley.wicks@newcastle.edu.au (L.K.M.-W.); 3 Rural Health School, College of Science, Health and Engineering, La Trobe University, Bendigo, VIC 3552, Australia; M.McEvoy@latrobe.edu.au; 4 Centre for Clinical Epidemiology & Biostatistics, Hunter Medical Research Institute, School of Medicine & Public Health, The University of Newcastle, Newcastle, NSW 2308, Australia; 5 Centre for Brain and Mental Health Research, Hunter Medical Research Institute, School of Medicine & Public Health, University of Newcastle, Newcastle, NSW 2308, Australia; peter.schofield@newcastle.edu.au

**Keywords:** diet quality, cognitive performance, Food Frequency Questionnaire (FFQ), Hunter Community Study (HCS), Australian Recommended Food Score (ARFS), Audio Recorded Cognitive Screen (ARCS), Mini Mental State Examination (MMSE)

## Abstract

There is a lack of evidence to determine if diet quality is associated with cognitive performance in older adults. Therefore, the aim of this study was to examine whether diet quality is associated with cognitive performance among older adults. A cross-sectional, secondary analysis of baseline data from the Hunter Community Study (HCS), comparing diet quality, measured using the Australian Recommended Food Score (ARFS), along with validated cognitive performance instruments the Audio Recorded Cognitive Screen (ARCS) and the Mini-Mental State Examination (MMSE) were undertaken in adults aged 55–85 years, living in Newcastle, NSW, Australia. Adjusted linear regression analyses showed that, compared with the lowest ARFS quintile, those in the highest quintile had an ARCS score 5.883 units greater (*p* < 0.001; R^2^ = 0.0098). Furthermore, when quintiles of ARFS score were tested against each ARCS sub-scale score, statistically significant associations were observed with the greatest effect for the Memory (β = 4.055; *p* = 0.001; R^2^ = 0.0065) and Attention (β = 4.136; *p* = 0.002; R^2^ = 0.0047) domains. No statistically significant associations were observed between quintiles of ARFS and MMSE score in the adjusted linear regression analyses. In conclusion, a positive association was observed between diet quality and cognitive performance within this sample of older Australian adults. Further investigation of the above association over time, when follow-up data becomes available, in longitudinal analysis is recommended.

## 1. Introduction

The rapid increase in the prevalence of cognitive impairment in the population including those with dementia is of global concern. Several large population-based studies have demonstrated that significant cognitive decline begins at around age 55 years [1,2]. According to a recent report, the estimated worldwide prevalence of dementia in the population over 60 years of age was 50 million people in 2018 [3]. It is estimated to rise 1.5 times by 2030, and to triple by 2050 [3]. The estimated total societal cost of dementia was around US$1 trillion in 2018, globally [3]. Alzheimer’s disease was the fourth leading cause of disability-adjusted life-years (DALYs) in the age group 75 years and older in 2019 worldwide [4]. It was the sixth leading cause of death in the US in 2019 [5]. According to a recent report from the Care Policy and Evaluation Centre, London School of Economics and Political Science, there were almost 885,000 old people with dementia in the United Kingdom (UK) and the prevalence rate was 7.1% in 2019 [6]. The estimated number of people living with dementia in Canada was 564,000 in 2016 and it was estimated to have increased to 937,000 in 2031 [7]. In 2016, the prevalence of dementia in Australia was estimated to be about 400,833 people, and it is expected to increase to 760,672 by 2036 and 1,100,890 by 2056 [8]. In 2016, dementia cost Australia more than $14 billion, which is equivalent to an estimated $35,550 per person with dementia [8]. Dementia has risen from the fourth leading cause of death for Australians in 2007 to the second in 2017, accounting for 8.5% of all deaths [9].

A decline in cognitive abilities, such as conceptual thinking, memory, and speed of processing, are considered part of the normal aging process [10]. Mild cognitive impairment (MCI) is described as a transitional state of subclinical cognitive decline, to an extent whereby it is greater than expected for that age [11,12], but does not yet meet currently accepted clinical criteria for Alzheimer’s Disease (AD) [11,12], the most common type of dementia [13].

Diet is one of the modifiable risk factors for cognitive impairment [14]. Dietary pat-terns, such as diets high in omega-3 polyunsaturated fatty acids (n-3 PUFAs) or vegetables or a Mediterranean diet (MeDi) could potentially prevent or delay cognitive decline or AD [15,16]. Diets high in fruits and vegetables are rich in antioxidants, which could be useful for both preventing and treating AD, by reducing oxidative stress that might con-tribute to the neurotoxic effect induced by amyloid-b in AD [17]. Several longitudinal cohort studies and a cross-sectional study showed an association between dietary vitamin E, vitamin C, flavonoids, and/or b-carotene and reduced rates of dementia and/or AD [18,19,20]. However, two longitudinal studies found that the antioxidants mentioned above had a negligible effect on the incidence of dementia or AD [21,22].

The relationship between diet and cognition has been mostly examined using a single-nutrient approach, and the results obtained are inconsistent [23]. Specific dietary pat-terns, which consist of a combination of nutrients, may be better at preventing cognitive decline [24], as interactions between nutrients may enhance the benefits of individual nutrients [25]. An a priori approach can be used to derive dietary patterns when studying the relationship between diet and cognitive status. Dietary patterns derived from an a priori approach relate to a score that shows how well a subject adhered to a predefined ‘healthy’ diet [23].

Several scores have been developed based on different countries’ dietary guidelines. The Healthy Diet Indicator (HDI), based on the World Health Organization dietary guide-lines to prevent chronic disease, was used in a cohort study by Leite et al. in 2001 [26]. It showed that a decrease in the prevalence of cognitive decline was linked to a higher HDI score [26]. The Recommended Food Score (RFS) by Kant et al. (2009), based on the Dietary Guidelines for Americans [27], was used in the Cache County study and showed a positive association between RFS scores and mean adjusted Modified Mini-mental State Examination (3MS) scores at baseline [28]. The Australian Recommended Food Score (ARFS) [29] is a brief food-based diet quality index that was adapted from the RFS [27] using the Australian Dietary Guidelines (ADG) [30] recommendations for Australian populations.

The Mini-Mental State Examination (MMSE), an interview-administered screening tool to assess global cognitive function, is the most frequently used tool to examine cognition. ARFS and Audio Recorded Cognitive Screen (ARCS), an objective self-administered clinical measure to identify distinct patterns of cognitive impairment, have not been used previously to examine the link between diet quality and cognition in adults aged 55–85 years. This study aims to evaluate whether diet quality is associated with cognitive performance in adults aged 55–85 years who participated in the Hunter Community Study (HCS) in Newcastle, Australia. It is hypothesized that men and women who have better diet quality, as evidenced by higher scores on ARFS, will have better cognitive performance, as determined by higher MMSE and ARCS scores. Given that diet quality has been shown to differ between men and women [31,32], it is also hypothesized that an association would differ according to gender.

## 2. Materials and Methods

### 2.1. Study Sample

The current study is a cross-sectional secondary analysis of data from the Hunter Community Study (HCS), a cohort study conducted as a collaboration between the Hunter New England Area Health Service and the University of Newcastle’s School of Medicine and Public Health [33]. Baseline data from men and women aged 55–85 years who resided in Newcastle, New South Wales (NSW), Australia were collected between 2004 and 2007. Participants were randomly selected from the NSW State electoral roll. Adults aged 55–85 years listed on the NSW electoral roll within Newcastle were eligible to participate. Persons who could not speak English and those living in a residential aged-care facility were deemed ineligible. All participants were required to complete self-administered questionnaires covering demographics, dietary intake, morbidity, mental health, quality of life and physical activity. A range of clinical measurements was also performed by a trained nurse in a clinic visit which included anthropometry, respiratory function, cardiovascular function, cognition, bone mineral density, functional capacity, and blood biomarkers. A total of 9784 individuals were sent invitation letters, 7575 responded (77.4%), 3877 agreed to participate via written informed consent, and 3253 (response rate 44.5%) completed the study (47% men and 53% women). Further details regarding recruitment have been published elsewhere [33]. The gender and marital status of participants approximately reflect the profile of the Hunter, state and national populations, but with a slightly lower mean age (i.e., 66.3 years) [33]. This study was conducted according to the guidelines laid down in the Declaration of Helsinki and all procedures involving research study participants were approved by the University of Newcastle Human Research Ethics committee (code: H-820–0504, date: February 2004). Written informed consent was obtained from all subjects/patients. ‘Memorandum of Understanding’ and ‘Confidentiality Statement’ were signed, and ‘2007 Application for Variation of Ethics Approval for research involving humans’ was submitted and approved.

### 2.2. Food Frequency Questionnaire (FFQ)

Dietary intake at baseline was measured by a self-administered 145-item semi-quantitative Food Frequency Questionnaire (FFQ), that was previously validated against four-day weighed food records in older Australian adults participating in the Blue Mountain Eye study [34]. The participants were required to indicate their usual frequency of consumption of food items, with a nine-category frequency scale ranging from ‘never’ to ‘four or more times per day’ during the previous 12 months [34].

### 2.3. Diet Quality Measures

The primary exposure variable for the study was diet quality, which was measured using the ARFS [29]. The ARFS score was calculated from the FFQ. The ARFS focuses on the variety of foods consumed within each recommended food group in the Australian Dietary Guidelines (ADG) [30]. It contains seven categories—vegetables, fruit, protein food, grains, dairy products, fats and alcohol [35]. One point is usually assigned for most of the foods if they were consumed at least once per week, depending on the ADG [30,36]. For example, FFQ items including fruits and vegetables consumed less than once a week scored zero and those consumed once a week or more scored one; meat FFQ items scored zero if consumed less than once a month or 5 or more times per week and scored 1 if consumed 1–4 times per week. Additional points were also awarded for the type and quantity of core food intake consistent with national dietary intake recommendations. A maximum of two points were added for alcohol consumption: one point for moderate frequency (up to 4 days/week) and the second point for moderate quantity (1 or 2 standard drinks, when alcohol was consumed). The ARFS score is the sum of each item, and the maximum score is 74 [35]. ARFS has been validated by comparing ARFS scores to dietary intake data derived using the Australian Eating Survey Food Frequency Questionnaire (AES FFQ) [35] collected over two administration rounds, five months apart. Correlations between the ARFS and the AES FFQ for all nutrients tested were significantly greater than 0.3. This indicated that the ARFS can be used to assess dietary patterns and evaluate associations between diet and health status [35]. The higher the ARFS score, the healthier the diet. More detailed explanation for calculating the ARFS has been described elsewhere [35].

### 2.4. Cognitive Performance Outcome Measures

The primary outcome variable for the study was cognitive performance. This was measured by the total score of the MMSE [37] and the ARCS [38]. The ARCS was used as an additional cognitive instrument as it also assesses several cognitive sub-domains, is more sensitive to fine grade changes in younger age, and, unlike the MMSE, is not subject to ceiling effects [38].

The MMSE is an interview-administered screening tool for detecting cognitive impairment [37]. There are two parts to the examination. The first part requires vocal responses only and examines orientation, memory and attention. The maximum score is 21. The second part assesses the participant’s ability to name objects, follow verbal and writ-ten commands, write a sentence and copy a figure, with a maximum score of 9 [37]. The total score is the sum of the scores for each part, which has a maximum of 30. A score ≤23 suggests cognitive impairment [37]. The MMSE has been validated in a sample of 206 patients with dementia, depression with cognitive impairment or various uncomplicated types of depression, and 63 normal subjects [37]. Concurrent validity was determined in the correlation of MMSE scores with the Wechsler Adult Intelligence Scale, Verbal and Performance scores—MMSE vs. Verbal IQ, Pearson r = 0.776 (*p* < 0.0001) and MMSE vs. Performance IQ, Pearson r = 0.660 (*p* < 0.001) [37]. The reliability of MMSE has also been tested, with positive results on both 24-h (Pearson r = 0.887) and 28-day retests (Pearson r = 0.988) [37].

The ARCS is an objective, self-administered clinical measure of cognitive performance [38]. It was developed to identify distinct patterns of cognitive impairment by ana-lysing the five key cognitive domains: memory, verbal fluency, visuospatial functioning, language and attention/executive function, and speed of writing [38]. An overall ARCS score is the sum of the scaled scores for each of the five domains, revised to a population mean of 100 and a standard deviation (SD) of 15 [38]. An ARCS score below 85 suggests cognitive impairment. ARCS has been validated against neuropsychological tests and showed good validity (mostly within the range of r = 0.50–0.70, *p* < 0.001) and reliability (range of r = 0.70–0.88) in measuring functioning in the multiple cognitive domains [38]. Construct validity has been supported by correlations between raw ARCS measures and conventional neuropsychological tests probing comparable cognitive domains [38].

### 2.5. Potentially Confounding Variables

Potential confounding variables were identified based on reviewing the literature and according to the classical definition of a confounding variable (i.e., association between ARFS and cognition score that is not an intermediate in the causal chain) [39]. The variables considered were age (years); gender; Body Mass Index (BMI) (weight in kg/height in m^2^); physical activity level (the mean number of steps per day); education level (completed year 10 or below; completed year 11, 12 or trade or technical college qualification; and University or other comparable tertiary qualification); smoking status (never smoked, ever smoked and current smoker); income (above or below $40,000 per year); alcohol intake (non-drinker, safe drinker, moderate drinker, hazardous drinker—binge, hazardous drinker—chronic [40]); multivitamin/mineral supplement use (yes or no); total energy intake (kJ per day); serum fasting glucose, cholesterol, triglyceride and C-reactive protein (mmol/L) and self-reported physician-diagnosed co-morbidities: diabetes, asthma, hyper-tension, heart attack and stroke (yes or no to represent presence) [41].

### 2.6. Statistical Analysis

Statistical analysis was completed using STATA software version 15 [42]. The linear relationship between each independent variable and the dependent variable were verified by visual inspection of scatterplots. The normality of the regression residuals was verified by visual inspection of histograms. In an available case analysis, the ARFS scores derived from FFQ data at baseline were tested for associations with cognitive performance that were recorded as either total score (i.e., MMSE and ARCS) or sub-score in individual cognitive domains (i.e., ARCS only, as no MMSE sub-scores were available). For analyses, participants were divided into quintiles based on their ARFS diet quality scores. The lowest quintile for ARFS diet quality score represents the reference category. Continuous variables were summarized using mean and standard deviation for normally distributed variables and median, minimum and maximum for variables that were not normally distributed. Categorical variables are presented as proportions. Baseline characteristics were summarized and compared across ARFS quintiles. A *p*-value <0.2 was considered statistically significant to determine potential confounders by testing the statistical association between cognitive performance measure and diet quality measure, based on the definition of a confounder [39]. Physical activity was measured by a Digiwalker SW-200 pedometer (Yamax Corp, Tokyo, Japan) which is a valid measure of free-living activity [43]. The pedometer was worn on the waist belt, in line with either leg during waking hours for 7 days. Participants recorded the start and end time for pedometer-wear each day, and step count each evening, but did not reset the counter. Days with less than 9 h of wear as indicated by the diary, and participants with less than 3 days of measurement, were excluded from the analysis [44]. Participants with missing ARFS and MMSE or ARCS data were excluded from the analysis.

The linear test for trend was performed for all variables and shown in Supplementary Table S1. Linear regression models were used to assess the association between ARFS and total MMSE and ARCS scores. Univariate linear regression was utilized to examine the association without adjustment for potential confounders. Multivariate linear regression was performed to assess the association between ARFS scores and MMSE total score, ARCS total score, and ARCS sub-scores with adjustment for potential confounders. The unadjusted and adjusted models are presented. The adjusted model included alcohol use, smoking, daily step count and total energy intake. The adjusted model included all individuals with available covariate data; hence the sample size is smaller due to missing covariate data for some individuals. Age, gender, and education were not included in the model as the ARCS score is already standardized by age, gender, and education. A *p*-value <0.05 was considered statistically significant and regression coefficients, R-squared, and *p*-values were reported as appropriate.

A sex-specific subgroup analysis (determined a priori) was performed to determine if there was an interaction effect of gender on the association between total ARFS score and total MMSE and total ARCS scores, respectively.

Given the cross-sectional design of this study, a sensitivity analysis was performed by excluding all participants with an MMSE score <24 or an ARCS score <85, to determine if the association between diet quality and cognitive performance was influenced by cognitive impairment within some of the study sample (i.e., reverse causation).

## 3. Results

### 3.1. Baseline Characteristics of Study Population

There were 3253 men and women who completed the baseline surveys for the HCS. From this, there were 2890 participants who also completed the FFQ questionnaire and had usable data. A total of 2125 participants (1029 men and 1096 women), aged 55–85 years (mean: 66.1 yrs, SD: 7.3 yrs), remained in the unadjusted analyses after omitting those participants with missing ARFS, MMSE or ARCS scores. Due to missing covariate data, there were 1795 and 1797 participants that remained in the adjusted analyses for MMSE and ARCS, respectively.

The HCS data was compared with the Hunter, state, and national populations. The gender and marital status of participants approximately reflect the profile of the Hunter, state, and national populations, but with a slightly lower mean age [33]. The socio-demo-graphic and baseline characteristics of the study population are reported in Table 1. Demographic data appear similar between genders, except for smoking status and alcohol intake, which were higher for men. Men appear slightly higher on household income and educational level than women. The proportion of men who had a heart attack appears higher than that of women. The descriptive statistics of the ARFS, MMSE and ARCS scores of the participants are described in Table 1. Approximately 26.3% of the study sample had a total ARCS score less than 85, suggestive of cognitive impairment. Scores for women on both MMSE and ARCS appear marginally higher than those for men in the study sample. The descriptive statistics of the sample dietary intake are also presented in Table 1.

### 3.2. Baseline Characteristics for Participants from the HCS According to ARFS Quintiles

Baseline characteristics for participants aged 55–85 years (n = 2125) from the HCS, ac-cording to ARFS quintiles, are reported in Supplementary Table S1. Participants in the highest quintile for ARFS diet quality score (Q5, ARFS ≥ 35) were more likely to be female, younger in age, have a higher mean number of steps per day, higher daily energy intake, higher household income, higher education level, lower serum triglyceride, have higher total MMSE score, higher total ARCS score or higher ARCS sub scores, i.e., memory, fluency and attention. Participants reporting higher ARFS diet quality scores were also less likely to smoke or drink alcohol in comparison to participants in the lowest ARFS diet quality scores (Q1, ARFS < 21).

### 3.3. Association between ARFS and MMSE Score

The association between quintiles of ARFS and MMSE score are reported in Table 2. In the unadjusted linear regression model, compared with the lowest ARFS quintile, those in the highest ARFS quintile had a higher MMSE score. In the adjusted model (adjusted for age, gender, education, alcohol use, smoking, daily step count and total energy), those in the highest ARFS quintile had a higher MMSE than those in the lowest ARFS quintile, but this was not statistically significant. No analysis is presented for MMSE subdomains as only total MMSE scores were available.

### 3.4. Association between ARFS and ARCS Total and Sub-Scale Scores

The association between quintiles of ARFS and ARCS total score and ARCS sub-scale scores are reported in Table 3. In adjusted linear regression analyses (adjusted for age, gender, education, alcohol use, smoking, daily step count and total energy), compared with the lowest ARFS quintile, those in the highest ARFS quintile had a higher ARCS score. There was also a statistically significant association between quintiles of ARFS and each ARCS sub-scale score with the greatest effect observed for the memory and attention do-mains.

### 3.5. Sex-Specific Subgroup Analysis

A subgroup analysis was performed to determine if there was an interaction effect of gender on the association between total ARFS score and total ARCS score. However, this was not statistically significant (*p* = 0.345) (results not shown). The effect of gender on the association between ARFS and total MMSE score was not performed as there was no overall effect observed.

### 3.6. Sensitivity Analysis

A sensitivity analysis was performed by excluding participants who had an ARCS score less than 85 or an MMSE score <24 to determine if there was reverse causation due to cognitive impairment. Approximately 26.3% of the study sample had a total ARCS score less than 85, suggestive of cognitive impairment. After removing these participants, in adjusted linear regression analyses, compared with the lowest ARFS quintile, those in the highest quintile had an ARCS score 2.396 units greater (*p* < 0.044). The absence of an association between ARFS and MMSE remained after removing those with an MMSE score <24 (Results not shown).

## 4. Discussion

This study aimed to evaluate whether diet quality is associated with cognitive performance in adults aged 55–85 years who participated in the HCS in Newcastle, Australia.

A statistically significant association across quintiles of ARFS score and total ARCS score after adjusting for confounding variables was identified (Table 3). Those in the highest total ARFS quintile had a significantly higher total ARCS score. Although the effect of diet quality on ARCS score was statistically significant, on a scale of 20 to 147 (range of ARCS score in HCS), a difference of approximately 6 units may not be clinically meaningful. Furthermore, the adjusted R^2^ for the adjusted model was below 1%. This suggests that less than 1% of the variation in the total ARCS score is explained by diet quality.

There was also a statistically significant association between each quintile of ARFS diet quality score and each ARCS sub-scale score, with the greatest effect observed for the memory and attention domains. This suggests that diet quality may be crucial for all components of cognition, with the greatest benefit observed for memory and attention. However, the absolute difference in total ARCS score between the highest and lowest ARFS quintiles was small and may not be clinically meaningful. Furthermore, the adjusted R^2^ for ARCS sub-domain scores was even smaller. There was no association across quintiles of ARFS score and MMSE score in this population. The ARCS tool was recently developed, so there is limited literature on its use. There has been no previous research using it to examine the association between diet and cognitive performance. A study by MacDonald-Wicks et al. (2019) identified statistically significant associations between n-6 fatty acid intakes and Fluency, Visual, Language, and Attention domains of ARCS [45]. However, the aforementioned study explored the relationship between specific nutrients and cognition rather than dietary patterns.

From the demographic, lifestyle and dietary characteristics of the study sample, on average the HCS participants appeared to be in overall good health and were eating a healthy diet. Compared with the results of the Australian Health Survey (AHS), the HCS sample had a higher proportion of non-smokers, higher daily intakes of carbohydrate, vitamin B12, calcium, zinc, fish, fruit, red meat and vegetables, and less daily fat and multi-vitamin supplement consumption than similar age groups in the AHS [46]. These differences are likely to be attributable to the different sociodemographic characteristics between AHS and HCS samples. For example, HCS did not include people aged over 85 years, as were included in the AHS, and only recruited participants in the Hunter region rather than nationwide. The mean MMSE score of this sample was 28.0 (SD: 1.5) and the mean score for total ARCS was 98.9 (SD: 15.9) which indicates that on average the study participants had good cognitive function, as a score below 23 for MMSE [37] and below 85 for ARCS suggests cognitive impairment [38].

To the best of the author’s knowledge, this is the first study to examine the association between diet quality using the ARFS and cognition measured using MMSE and ARCS in an older Australian population. There have been several cross-sectional and cohort studies that have examined the association between diet quality, measured using different diet quality indices that assess against national or international dietary guidelines similar to ARFS, and cognitive performance measured using MMSE or other cognitive tests, on older men and women, but there are no consistent results from these studies [26,28,47,48,49].

The fact that the current study shows no statistically significant association between quintiles of ARFS score and MMSE score could be due to two main reasons. Firstly, the MMSE has well known ceiling effects that are likely to be most prominent in younger people in whom cognitive disorders are less common. The ARCS has no ceiling effects as performance on several of the component tests that contribute to the total ARCS score (specifically, the verbal fluency tasks) have no maximum possible score [38]. The positive findings with ARCS and negative findings with MMSE are likely to reflect these differences in properties of the cognitive test instruments. Secondly, the MMSE has been used in many studies to examine cognitive deficit, especially in longitudinal analyses, as it is good at measuring changes in cognitive function over time [50]. It may not be sensitive enough to detect differences in cognitive performance in a cross-sectional design, especially of a mostly cognitively well cohort. It may also be more sensitive in a sample that already has significantly impaired cognition; the HCS sample had relatively good cognitive function.

Although there was a statistically significant association between ARFS score and ARCS score, the R^2^ for the regression was small, and this may be due to the relatively good health of the study sample and the cross-sectional study design. However, these statistically significant results should provide direction for further longitudinal research.

The mean ARFS score for the HCS sample was quite low. However, the number of servings of vegetables and fruits met the Australian Guide for Healthy Eating (AGHE) recommended daily serves, i.e., 5–5.5 servings of vegetables and 2 servings of fruits for men and women aged over 50 [51]. This reflects a limitation of the ARFS, which does not incorporate the frequency of consumption of fruits and vegetables, as only 1 point can be allocated for each variety consumed per week. HCS participants reported having adequate servings of fruits and vegetables on the FFQ, but this is likely to have included limited variety on a weekly basis.

A strength of this study lies in its novelty, with no similar studies performed in this region. The study sample size was large and there was the ability to adjust for multiple confounding variables. Validated dietary assessment and cognitive performance tools were used, the MMSE and ARCS are well understood and have cut-off points to indicate cognitive impairment (23 [37] and 85 [38] respectively). The findings of the current study are limited because of missing data due to a lack of cognitive assessment measures in every participant who completed the study surveys, including the dietary assessment; however, there was still sufficient statistical power to detect the hypothesized association. Most study participants were relatively well and had good cognitive function with an ARCS score greater than 85. Results from cognitively impaired individuals may be less re-liable as under- or over-estimation may occur, due to difficulty in recalling the type, amount and frequency of food intake. Another limitation is that the sample used for this analysis has a slightly lower mean age than the Hunter, state, and national populations so these results may not be completely representative of the general 55- to 85-year-old population. Due to the cross-sectional study design, causation between the ARFS and cognition cannot be inferred. To determine if this was influencing the results, a sensitivity analysis excluding people with a total ARCS score of less than 85, suggestive of cognitive impairment, was performed. Although the effect of ARFS on ARCS score was attenuated, it remained statistically significant, suggesting that the main findings are robust. However, given that no formal neuropsychological test battery was used for dementia, some people with cognitive impairment may have been missed. There is always the potential for measurement error, as a self-reported FFQ was used; however, the Blue Mountains Eye Study FFQ is validated for use within an older cohort, though it has not been validated specifically against the ARFS. Data for each of the food groups were realistic when compared with the AHS (2011–12). For further investigations, longitudinal analysis is recommended to assess differences in cognitive function over time when follow-up data become available.

## 5. Conclusions

The present study showed that better diet quality was associated with better cognitive performance, determined using the ARCS, among a sample of older Australian adults, though the effect size was small in this cross-sectional study. Further investigation of the association between diet quality and cognitive function or decline over time, when follow-up data become available, in longitudinal analysis is recommended.

## Figures and Tables

**Table 1:** Baseline characteristics for participants in the Hunter Community Study cohort of men and women aged 55–85 years.

Variables	Unit of Measurement	Men (n = 1029)			Women (n = 1096)			Total (n = 2125)		
		N	Mean (SD)	Median (Min, Max)	N	Mean (SD)	Median (Min, Max)	N	Mean (SD)	Median (Min, Max)
Age at baseline	Year	1029	66.5 (7.5)		1092	65.6 (7.1)		2121	65 (7.3)	
BMI ^1^	Wt in kg/ht^2^ in m^2^	1028	28.7 (4.1)		1094	28.7 (5.6)		2122	28.7 (4.9)	
Physical activity level	Mean no. of steps/day	939		6261.1 (191.7, 21151.3)	1014		6853.8 (275.5, 17311.4)	1953		6550.1 (191.7, 21151.3)
Serum fasting glucose	mmol/L	847	5.3 (1.4)		908	4.9 (0.9)		1755	5.1 (1.2)	
Serum cholesterol	mmol/L	958	4.8 (1.0)		1008	5.3 (1.0)		1966	5.1 (1.0)	
Serum triglyceride	mmol/L	954		1.2 (0.3, 12.7)	1008		1.1 (0.2, 9.8)	1962		1.2 (0.2, 12.7)
C-reactive Protein	mmol/L	877		1.9 (0.4, 45.5)	879		2.2 (0.4, 103.1)	1756		2 (0.4, 103.1)
Energy	kJ/day	1029		8210.0 (2688.8, 26127.8)	1096		7434.3 (0, 35492.3)	2125		7803.4 (0, 35492.3)
Vegetables	serve/day ^2^	1029		5.1 (1.0, 31.7)	1094		5.5 (0.9, 46.9)	2123		5.2 (0.9, 46.9)
Fruit	serve/day ^3^	1029		1.6 (0, 18.8)	1094		2.1 (0, 31.3)	2123		1.9 (0, 31.3)
Red meat	g/day	1029		66.2 (0, 455)	1094		57.3 (0, 1107.6)	2123		62.2 (0, 1107.6)
Chicken	g/day	1029		15.3 (0, 120.5)	1094		17.3 (0, 383.3)	2123		15.3 (0, 383.3)
Fish	g/day	1029		22.3 (0, 329.5)	1094		24.7 (0, 650.7)	2125		24.1 (0, 650.7)
ARFS ^4^ Score		1029	26.9 (8.0)		1096	29.5 (7.9)		2125	28.2 (8.1)	
MMSE ^5^ Score		1029	27.8 (1.6)		1096	28.2 (1.5)		2125	28.0 (1.5)	
ARCS ^6^ Score		1029	98.1 (16.2)		1096	99.5 (15.7)		2125	98.8 (15.9)	
ARCS subgroup -										
Memory		1029	99.4 (16.2)		1096	101.1 (14.3)		2125	100.3 (15.3)	
Fluency		1029	97.7 (13.8)		1096	99.2 (14.4)		2125	98.5 (14.1)	
Language		1029		106 (0, 115)	1096		103 (0, 116)	2125		103 (0, 116)
Attention		1029	100.9 (16.2)		1096	98.7 (16.3)		2125	99.8 (16.3)	
Visuospatial		1029		103 (29, 121)	1096		101 (31, 123)	2125		103 (29, 123)

^1^ BMI, Body Mass Index; ^2^ A standard serving of vegetables is about 75 g; ^3^ A standard serving of fruits is about 150 g; ^4^ ARFS, Australian Recommended Food Score; ^5^ MMSE, Mini-Mental State Examination; ^6^ ARCS, Audio Recorded Cognitive Screen.

**Table 2:** Unadjusted and adjusted linear regression of MMSE score (n = 2125) by quintiles of ARFS score in the Hunter Community Study.

Variable		N	ARFS ^1^									Adjusted R^2^
			Quintile 1 (<21)	Quintile 2 (21–25)		Quintile 3 (26–29)		Quintile 4 (30–34)		Quintile 5 (≥35)		
				Coefficient	*p*-Value	Coefficient	*p*-Value	Coefficient	*p*-Value	Coefficient	*p*-Value	
**MMSE ^2^**	Unadjusted	2125	1.0 [Reference]	**0.266**	**0.015**	**0.314**	**0.004**	**0.302**	**0.005**	**0.263**	**0.014**	0.0033
	Adjusted ^3^	1795	1.0 [Reference]	0.079	0.497	0.121	0.304	0.072	0.544	0.004	0.976	0.0567

^1^ ARFS, Australian Recommended Food Score; ^2^ MMSE, Mini-Mental State Examination; ^3^ Adjusted model, Multivariable analyses confounder adjusted age, gender, education, alcohol use, smoking, daily steps count, total energy; Bold: Statistically significant (*p* < 0.05).

**Table 3:** Unadjusted and adjusted linear regression of total ARCS score (n = 2125) and ARCS subdomain scores by quintiles of ARFS score in the Hunter Community Study.

Variable		N	ARFS ^1^									Adjusted R^2^
			Quintile 1 (<21)	Quintile 2 (21–25)		Quintile 3 (26–29)		Quintile 4 (30–34)		Quintile 5 (≥35)		
				Coefficient	*p*-Value	Coefficient	*p*-Value	Coefficient	*p*-Value	Coefficient	*p*-Value	
**Total ARCS ^2^ score**	Unadjusted	2125	1.0 [Reference]	1.527	0.181	2.132	0.062	1.225	0.271	**3.787**	**0.001**	0.0042
	Adjusted ^3^	1797	1.0 [Reference]	1.921	0.118	**2.928**	**0.018**	2.126	0.084	**5.883**	**<0.001**	0.0098
**Memory domain**	Unadjusted	2125	1.0 [Reference]	−0.541	0.621	1.520	0.165	0.404	0.704	**2.150**	**0.044**	0.0024
	Adjusted	1797	1.0 [Reference]	−0.023	0.985	2.591	0.031	1.890	0.114	**4.055**	**0.001**	0.0065
**Fluency domain**	Unadjusted	2125	1.0 [Reference]	**2.749**	**0.006**	**3.000**	**0.003**	1.127	0.252	**3.540**	**<0.001**	0.0065
	Adjusted	1797	1.0 [Reference]	1.932	0.082	**2.731**	**0.014**	0.517	0.641	**3.510**	**0.003**	0.0077
**Language domain**	Unadjusted	2125	1.0 [Reference]	0.168	0.895	−0.165	0.897	−0.986	0.429	0.975	0.434	−0.0005
	Adjusted	1797	1.0 [Reference]	1.507	0.279	1.403	0.316	1.409	0.312	**3.575**	**0.014**	0.0025
**Attention domain**	Unadjusted	2125	1.0 [Reference]	1.424	0.224	1.751	0.135	2.054	0.072	**3.105**	**0.007**	0.0018
	Adjusted	1797	1.0 [Reference]	1.620	0.211	2.114	0.104	1.339	0.302	**4.136**	**0.002**	0.0047
**Visuospatial domain**	Unadjusted	2125	1.0 [Reference]	0.920	0.412	0.551	0.623	1.122	0.304	2.034	0.063	−0.0000
	Adjusted	1797	1.0 [Reference]	0.904	0.462	0.263	0.831	1.363	0.267	**3.044**	**0.018**	0.0042

^1^ ARFS, Australian Recommended Food Score; ^2^ ARCS, Audio Recorded Cognitive Screen; ^3^ Adjusted model, Multivariable analyses confounder adjusted age, gender, education, alcohol use, smoking, daily steps count, total energy; Bold: Statistically significant (*p* < 0.05).

## Data Availability

The final study dataset will be available to study investigators, Steering Committee members and the Research Ethics Boards if requested.

## Supplementary Materials

The following are available online at https://www.mdpi.com/2072-6643/13/3/909/s1, Table S1: Baseline characteristics for participants aged 55–85 years (n = 2125) from the Hunter Community Study according to ARFS quintiles.
