# Supplementary material for: Diet Quality and Cognitive Performance in Australian Adults Aged 55–85 Years: A Cross-Sectional Analysis of the Hunter Community Study Cohort
Source: Nutrients. 2021 Mar 11;13(3):909. doi: 10.3390/nu13030909 (PMC8000829; doi:10.3390/nu13030909)
Supplement: Supplementary file 1 [file nutrients-13-00909-s001.pdf]

Supplementary material

Table S1. Baseline characteristics for participants aged 55-85 years (n=2125) from the Hunter Community Study according to ARFS quintiles.

**Variables**	**Unit of measurement**	**ARFS[1] (Range)**																	**p-value**
		**Total (4-55)**			**Quintile 1 (<21)**			**Quintile 2 (21-25)**			**Quintile 3 (26-29)**			**Quintile 4 (30-34)**			**Quintile 5 (≥35)**		
		N	Mean	SD	N	Mean	SD	N	Mean	SD	N	Mean	SD	N	Mean	SD	N	Mean	SD	
Age at baseline	Year	2121	66.1	7.3	364	66.6	7.5	415	66.7	7.8	414	66.4	7.5	465	65.4	7.0	463	65.5	6.8	**0.003**
BMI[2]	Wt in kg/ ht^2 in m^2	2122	28.7	4.9	363	28.7	4.6	413	28.5	4.7	414	29.3	5.2	467	28.6	5.1	465	28.5	5.0	0.476
Physical activity level	Mean no. of steps/day	1953	6764.9	3156.6	330	6024.2	3112.5	381	6559.8	3117.1	379	6843.7	3127.5	427	6968.0	3142.7	436	7237.4	3163.0	**<0.001**
Energy	kJ/day	2125	8145.3	2727.14	364	6698.4	2189.3	415	7441.1	2218.0	414	7756.0	2078.2	467	8459.7	2215.2	465	9937.2	3405.1	**<0.001**
Serum fasting glucose	mmol/L	1755	5.1	1.2	288	5.2	1.3	350	5.1	1.0	351	5.1	1.1	377	5.0	1.2	389	5.1	1.3	0.206
Serum cholesterol	mmol/L	1966	5.1	1.0	325	5.0	1.1	383	5.0	1.0	393	5.1	1.0	430	5.1	1.0	435	5.1	1.0	0.259
Serum triglyceride	mmol/L	1962	1.4	0.9	323	1.5	0.9	382	1.5	1.0	393	1.4	1.0	429	1.3	0.8	435	1.2	0.7	**<0.001**
C-reactive Protein	mmol/L	1756	3.4	4.8	291	3.3	3.2	343	4.0	7.4	356	3.5	4.7	383	3.1	3.3	383	3.2	4.4	0.150
MMSE[4] Score		2125	28.0	1.5	364	27.7	± 1.6	415	28.0	1.6	414	28.1	1.5	467	28.0	± 1.5	465	28.0	1.4	**0.036**
ARC S[5] Score		2125	98.8	15.9	364	97.0	16.0	415	98.5	16.7	414	99.0	16.4	467	98.2	15.3	465	100.8	15.2	**0.003**
ARCS subgroup-																				
Memory		2125	100.3	15.3	364	99.5	15.9	415	99.0	16.3	414	101.1	14.2	467	99.9	15.1	465	101.7	14.8	**0.023**
Fluency		2125	98.5	14.1	364	96.3	14.2	415	99.1	14.1	414	99.3	14.6	467	97.5	13.8	465	99.9	13.7	**0.021**
Language		2125	97.9	17.8	364	97.9	17.3	415	98.1	17.2	414	97.8	19.4	467	96.9	17.7	465	98.9	17.4	0.715
Attention		2125	99.8	16.3	364	98.0	16.2	415	99.4	16.6	414	99.8	15.8	467	100.1	15.9	465	101.1	16.9	**0.008**
Visuospatial		2125	98.4	15.6	375	97.4	16.7	415	98.3	16.1	414	98.0	16.4	467	98.5	15.0	465	99.4	14.1	0.074

		Freq.	%	Freq.	%	Freq.	%	Freq.	%	Freq.	%	Freq.	%	
Gender	Male	1029	48.4	230	63.2	215	51.8	190	45.9	217	46.5	177	38.1	**<0.001**
Household income	> $40, 000 /year	821	41.3	111	33.0	146	38.0	176	44.7	192	44.3	196	44.6	**<0.001**
Education	Year 10 max	958	45.1	183	50.3	207	63.4	192	46.4	196	42.0	180	38.7	**<0.001**
	Year 11, 12 or TAFE	784	36.9	140	38.5	145	34.9	145	35.0	175	37.5.	179	38.5	
	University	383	18.0	41	11.3	63	15.2	77	18.6	96	20.6	106	22.8	
Smoke	Never	1155	54.8	166	46.2	221	53.5	217	52.5	270	59.0	281	60.7	**<0.001**
	Ever	787	37.4	133	37.0	161	39.0	171	41.4	162	35.4	160	34.6	
	Now	149	7.1	54	15.0	27	6.5	23	5.6	25	5.5	20	4.3	
	Unknown	15	0.7	6	1.7	4	1.0	2	0.5	1	0.2	2	0.4	
Alcohol use	Non-drinker	323	16.6	66	20.6	64	17.1	67	17.3	61	14.4	64	14.8	**0.002**
	Safe drinker	1232	63.3	177	53.4	232	61.0	243	62.8	284	66.8	302	69.9	
	Moderate drinker	154	7.9	22	6.9	32	8.4	33	8.5	29	6.8	38	8.8	
	Hazardous drinker – binge	123	6.3	29	9.0	325	6.6	29	7.5	26	6.1	14	3.2	
	Hazardous drinker – chronic	113	5.8	32	10.0	27	7.1	15	3.9	25	5.9	14	3.2	
Multivitamin supplement	Yes	234	11.2	45	12.7	45	11.0	43	10.5	41	9.0	60	13.1	0.959
Diabetes	Yes	214	10.3	39	11.0	50	12.3	40	9.8	40	8.8	45	9.9	0.238
Asthma	Yes	278	13.4	44	12.4	52	12.8	55	13.5	62	13.7	65	14.4	0.379
Hypertension	Yes	959	46.2	167	47.2	190	46.6	192	47.2	194	42.8	216	47.7	0.764
Heart attack	Yes	149	7.2	43	12.2	28	6.9	27	6.6	28	6.2	23	5.1	**0.001**
Stroke	Yes	67	3.2	15	4.2	16	3.9	12	3.0	15	3.3	9	2.0	0.063

Abbreviations:

1. ARFS, Australian Recommended Food Score, 2. BMI, Body Mass Index, 3. Alcohol use classified according to NHMRC guideline[35], 4. MMSE, Mini-Mental State Examination, 5. ARCS, Audio Recorded Cognitive Screen

Bold: Statistically significant ($p<0.05$)
